# Cells producing residual viremia during antiretroviral treatment appear to contribute to rebound viremia following interruption of treatment

**DOI:** 10.1371/journal.ppat.1008791

**Published:** 2020-08-25

**Authors:** Hadega A. Aamer, Jan McClure, Daisy Ko, Janine Maenza, Ann C. Collier, Robert W. Coombs, James I. Mullins, Lisa M. Frenkel

**Affiliations:** 1 Center for Global Infectious Disease Research, Seattle Children’s Research Institute, Seattle, Washington, United States of America; 2 Department of Global Health, University of Washington, Seattle, Washington, United States of America; 3 Department of Laboratory Medicine, University of Washington, Seattle, Washington, United States of America; 4 Department of Medicine, University of Washington, Seattle, Washington, United States of America; 5 Department of Microbiology, University of Washington, Seattle, Washington, United States of America; 6 Department of Pediatrics, University of Washington, Seattle, Washington, United States of America; Vaccine Research Center, UNITED STATES

## Abstract

During antiretroviral therapy (**ART**) that suppresses HIV replication to below the limit-of-quantification, virions produced during ART can be detected at low frequencies in the plasma, termed residual viremia (**RV**). We hypothesized that a reservoir of HIV-infected cells actively produce and release virions during ART that are potentially infectious, and that following ART-interruption, these virions can complete full-cycles of replication and contribute to rebound viremia. Therefore, we studied the dynamics of RV sequence variants in 3 participants who initiated ART after ~3 years of infection and were ART-suppressed for >6 years prior to self-initiated ART-interruptions. Longitudinal RV C2V5*env* sequences were compared to sequences from pre-ART plasma, supernatants of quantitative viral outgrowth assays (**QVOA**) of cells collected during ART, post-ART-interruption plasma, and ART-re-suppression plasma. Identical, “putatively clonal,” RV sequences comprised 8–84% of sequences from each timepoint. The majority of RV sequences were genetically similar to those from plasma collected just prior to ART-initiation, but as the duration of ART-suppression increased, an increasing proportion of RV variants were similar to sequences from earlier in infection. Identical sequences were detected in RV over a median of 3 years (range: 0.3–8.2) of ART-suppression. RV sequences were identical to pre-ART plasma viruses (5%), infectious viruses induced in QVOA (4%) and rebound viruses (5%) (total n = 21/154 (14%) across the 3 participants). RV sequences identical to ART-interruption “rebound” sequences were detected 0.1–7.4 years prior to ART-interruption. RV variant prevalence and persistence were not associated with detection of the variant among rebound sequences. Shortly after ART-re-suppression, variants that had been replicating during ART-interruptions were detected as RV (n = 5). These studies show a dynamic, virion-producing HIV reservoir that contributes to rekindling infection upon ART-interruption. The persistence of identical RV variants over years suggests that a subpopulation of HIV-infected clones frequently or continuously produce virions that may resist immune clearance; this suggests that cure strategies should target this active as well as latent reservoirs.

## Author summary

HIV-infected individuals receiving effective antiretroviral treatment (**ART**) produce virions detected in the blood at very low levels, termed residual viremia (**RV**). To understand the significance of RV as related to the persistence of HIV infection, we characterized the dynamics of RV sequence variants among plasma viruses over nearly a decade of ART and assessed whether RV contributed to rekindling viremia upon ART-interruption. The HIV reservoir producing RV appeared to be “seeded” at various times before ART-initiation. Identical RV sequences likely produced by a clonal cell population, varied over time, with unique sequence variants persisting over a median of 3 years. A subset of RV variants (14%) were identical to viruses found in pre-ART plasma, infectious viruses induced from cultured CD4^+^ T blood lymphocytes collected during ART, or in rebound plasma during ART-interruption. The persistence of unique RV variants over years, infers that the clones of HIV-infected cells producing these virions resist immune clearance or a subset of these clones are activated on a rolling basis, and that novel treatment strategies are needed to target this active reservoir that contributes to viral rebound.

## Introduction

Antiretroviral therapy (**ART**) has modified the course of human immunodeficiency virus type-1 (**HIV**) infection from a largely fatal disease to a chronic, treatable infection with a near-normal lifespan [1, 2]. Treatments for HIV infection continue to improve, with less frequent dosing of medicines and fewer adverse reactions. However, despite individuals having HIV replication suppressed by ART for decades, ART does not cure the infection [3, 4]. If ART is interrupted, infectious viruses resume replication. Given that cells capable of producing infectious viruses must be eliminated to cure HIV, many studies have focused on understanding the establishment of this reservoir [5–8] and the mechanisms that allow it to persist [9–12]. Studies have revealed essentially stable levels of HIV DNA in the blood [13]; although viral variants within the HIV DNA reservoirs appear dynamic [14], and clonally expanded infected cells increase in proportion over time [10, 15]. Most infected cells harbor defective proviruses [16–24], and during effective ART, cells with infectious proviruses appear to progressively diminish in frequency [25, 26], presumably due to lytic infection or elimination by cytotoxic-T-lymphocytes (**CTL**). An exception includes intact proviruses integrated in regions of the genome that are rarely transcribed [26, 27], which are hypothesized to be “deeply” latent. Our findings suggest that another exception may be clones harboring infectious variants that despite the production of residual viruses, resist immune clearance and contribute to rebound viremia.

During ART-suppression, not all proviruses persist in a latent state. Among infected individuals with plasma HIV RNA levels below the limit-of-quantification of clinical assays (<20–50 copies per milliliter (**c/mL**)), virions are produced (mean 1.5 copies per milliliter), termed residual viremia (**RV**) [28, 29]. RV are of interest as these arise from proviruses genetically-linked to replication-competent variants from quantitative viral outgrowth assays (**QVOA**) [30, 31], and as reported here, may contribute to rebound viremia. Analyses of RV, therefore, serve as a “window” to a segment of the reservoir capable of producing infectious virions, including variants that may escape clearance by lytic infection or immune elimination.

While current treatment regimens do not prevent the production of virions [28], ART blocks virions from infecting additional cells [15, 29, 32, 33]. Low-levels of HIV replication during ART can be detected by genetic divergence of regions of HIV *env* and/or *pol* sequences [29], regions that are under immune and drug selection, respectively. RV sequences generally do not diverge from those found immediately pre-ART [28, 34], consistent with suppression of full cycles of virus replication. While unique RV sequence variants have been observed to change in prevalence over time [14, 30], RV include viral variants with identical sequences within and across time points [30, 31, 35]. The detection of an identical RV variant as a majority variant (>50% of total RV sequences) and at multiple timepoints over 2.8 years of ART [14, 30] supports the hypothesis that RV are produced by proliferating clones. Biologic observations suggest that a single cell is unlikely to produce RV with identical sequences across multiple timepoints. An HIV infected cell undergoing lytic infection is estimated to release 50K virions [36], which could populate an individual’s ~3L of plasma or ~11L of extracellular fluid with 17 or 4.5 virions/mL, respectively; and since a virion contains two copies of the HIV genome, twice this number would be expected in plasma diluted to single viral templates. However, lytic infection destroys the cell. Thus, as clones of HIV infected cells are known to exist [10, 11, 17, 22], we reason that identical sequences, especially those detected across timepoints, likely come from clonal cell populations. We contend that RV arise from a subset of cells infected before the time of ART-initiation, which have been shown to proliferate and create clonal cell populations that persists during ART [32, 33].

The recognition that HIV-infected cells that proliferate during ART [10, 11, 15, 24, 29] produce infectious RV [30, 31] emphasizes that these clones are a barrier to curing HIV infection. To better understand this “active” HIV reservoir that persists and produces virions during ART-suppression, our study focused on the length of time that RV with identical sequences were produced, and the genetic linkage of these RV to sequence variants induced in QVOA of peripheral blood mononuclear cells (**PBMC**) collected during ART-suppression, or to plasma variants detected prior to ART-suppression or following ART-interruption. We hypothesized that HIV infected cells that persist during ART-suppression and contribute to RV are clonal and include infectious virus by virtue of detection of genetically identical variants across time, both within QVOA and within rebound viremia following ART-interruption.

## Results

**Fig 1:**
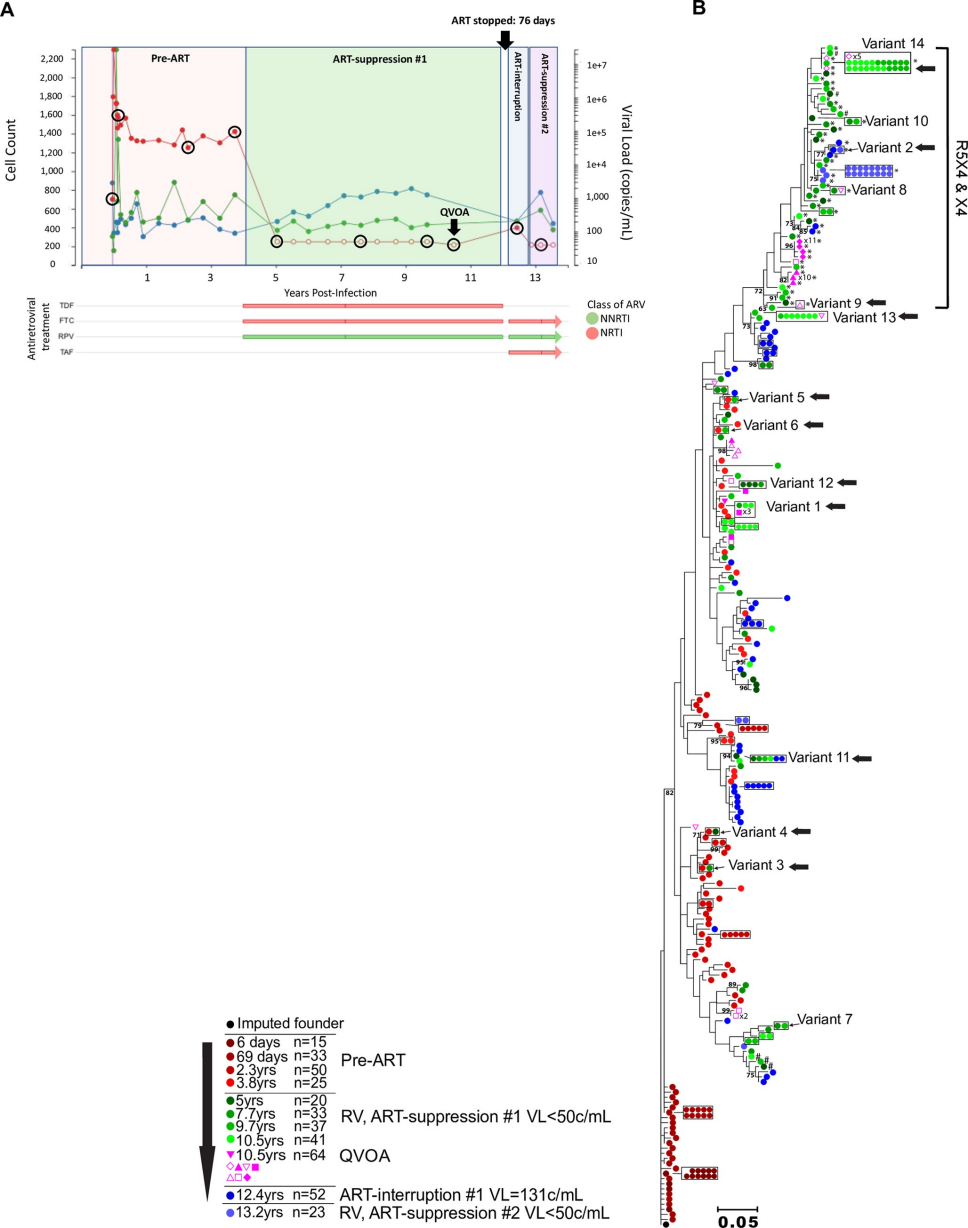
Clinical, antiretroviral treatment and phylogenetic tree for Participant 1. Plasma HIV RNA values (red), CD4^+^ (blue), and CD8^+^ (green) T-cell counts, antiretroviral treatment (x-axis) and phylogenetic analyses are shown in separate panels (**A,B**). Plasma HIV RNA symbols are filled when HIV RNA was detected and are open circles when below the lower-limit-of-quantification (either 40 or 50c/mL, depending on clinical assay employed). Timepoints selected for C2V5*env* sequencing from plasma have viral load symbols encircled in black, with the QVOA timepoint indicated by a black arrow. Antiretroviral treatments and time intervals prescribed are shown by horizontal bars at the bottom. The durations of self-initiated ART-interruptions are indicated by black arrows at top of each panel. Maximum likelihood phylogenetic trees were rooted with the consensus sequence of each participant’s predicted founder virus (consensus sequence of 1^st^ pre-ART timepoint). The key indicates the color code for the timepoints that specimens were collected relative to the estimated date of HIV infection, the antiretroviral treatment status, and the number of sequences derived from plasma or QVOA. Viral sequences from each unique QVOA well are represented by different symbols. Identical sequences are shown laterally and boxed for clarity. Significantly G-A hypermutated sequences are noted by #. CXCR4 and dual-tropic (R5X4 and X4) sequences are noted by *. Remaining sequences are predicted to be CCR5 (R5)-tropic. Residual viremia (RV) sequences identical to sequences from pre-ART plasma, viruses induced from QVOA cultures, or ART-interruption are shown with a horizontal black arrow. Clades with bootstrap values >70 are shown. Abbreviations: ARV: antiretroviral; AZT: zidovudine; DDI: didanosine; EFV: efavirenz; FTC: emtricitabine; 3TC: lamivudine; IDV: indinavir; NNRTI: non-nucleoside reverse transcriptase inhibitor; NRTI: nucleoside reverse transcriptase inhibitor; RLP: rilpivirine; D4T: stavudine; TAF: tenofovir alafenamide; TDF: tenofovir disoproxil.

**Fig 2:**
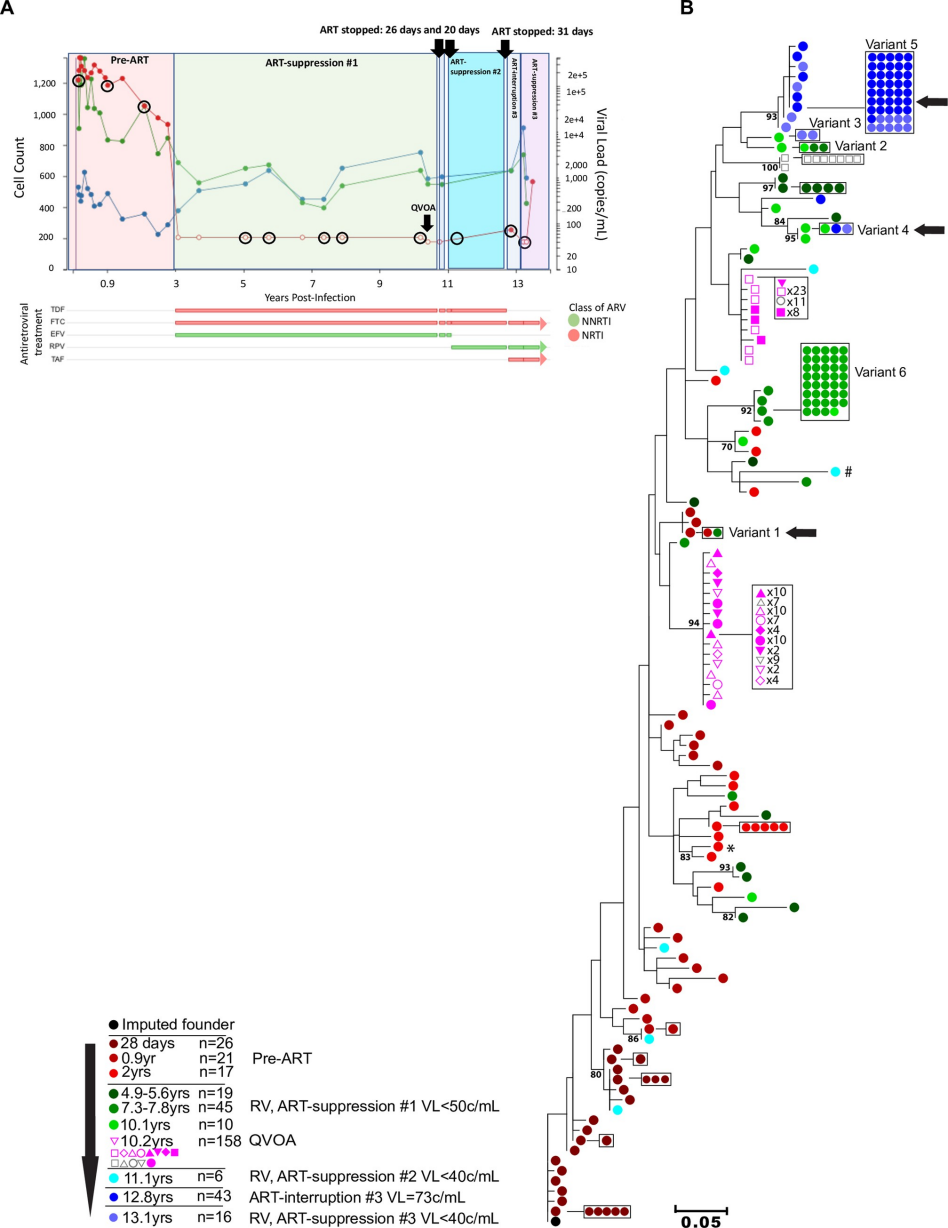
Clinical, antiretroviral treatment and phylogenetic tree for Participant 2. Plasma HIV RNA values (red), CD4^+^ (blue), and CD8^+^ (green) T-cell counts, antiretroviral treatment (x-axis) and phylogenetic analyses are shown in separate panels (**A,B**). Plasma HIV RNA symbols are filled when HIV RNA was detected and are open circles when below the lower-limit-of-quantification (either 40 or 50c/mL, depending on clinical assay employed). Timepoints selected for C2V5*env* sequencing from plasma have viral load symbols encircled in black, with the QVOA timepoint indicated by a black arrow. Antiretroviral treatments and time intervals prescribed are shown by horizontal bars at the bottom. The durations of self-initiated ART-interruptions are indicated by black arrows at top of each panel. Maximum likelihood phylogenetic trees were rooted with the consensus sequence of each participant’s predicted founder virus (consensus sequence of 1^st^ pre-ART timepoint). The key indicates the color code for the timepoints that specimens were collected relative to the estimated date of HIV infection, the antiretroviral treatment status, and the number of sequences derived from plasma or QVOA. Viral sequences from each unique QVOA well are represented by different symbols. Identical sequences are shown laterally and boxed for clarity. Significantly G-A hypermutated sequences are noted by #. CXCR4 and dual-tropic (R5X4 and X4) sequences are noted by *. Remaining sequences are predicted to be CCR5 (R5)-tropic. Residual viremia (RV) sequences identical to sequences from pre-ART plasma, viruses induced from QVOA cultures, or ART-interruption are shown with a horizontal black arrow. Clades with bootstrap values >70 are shown. Abbreviations: ARV: antiretroviral; AZT: zidovudine; DDI: didanosine; EFV: efavirenz; FTC: emtricitabine; 3TC: lamivudine; IDV: indinavir; NNRTI: non-nucleoside reverse transcriptase inhibitor; NRTI: nucleoside reverse transcriptase inhibitor; RLP: rilpivirine; D4T: stavudine; TAF: tenofovir alafenamide; TDF: tenofovir disoproxil.

**Fig 3:**
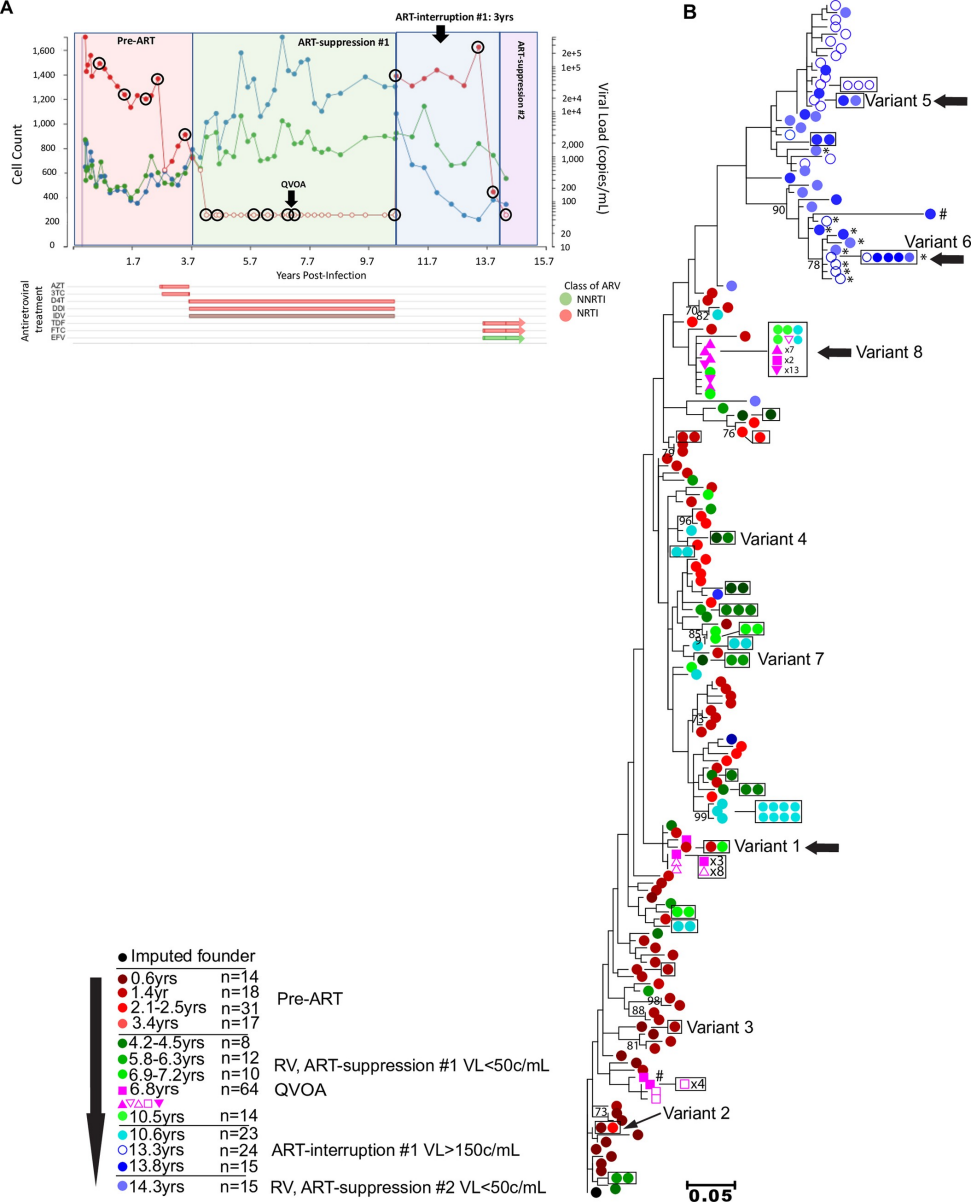
Clinical, antiretroviral treatment and phylogenetic tree for Participant 3. Plasma HIV RNA values (red), CD4^+^ (blue), and CD8^+^ (green) T-cell counts, antiretroviral treatment (x-axis) and phylogenetic analyses are shown in separate panels (**A,B**). Plasma HIV RNA symbols are filled when HIV RNA was detected and are open circles when below the lower-limit-of-quantification (either 40 or 50c/mL, depending on clinical assay employed). Timepoints selected for C2V5*env* sequencing from plasma have viral load symbols encircled in black, with the QVOA timepoint indicated by a black arrow. Antiretroviral treatments and time intervals prescribed are shown by horizontal bars at the bottom. The durations of self-initiated ART-interruptions are indicated by black arrows at top of each panel. Maximum likelihood phylogenetic trees were rooted with the consensus sequence of each participant’s predicted founder virus (consensus sequence of 1^st^ pre-ART timepoint). The key indicates the color code for the timepoints that specimens were collected relative to the estimated date of HIV infection, the antiretroviral treatment status, and the number of sequences derived from plasma or QVOA. Viral sequences from each unique QVOA well are represented by different symbols. Identical sequences are shown laterally and boxed for clarity. Significantly G-A hypermutated sequences are noted by #. CXCR4 and dual-tropic (R5X4 and X4) sequences are noted by *. Remaining sequences are predicted to be CCR5 (R5)-tropic. Residual viremia (RV) sequences identical to sequences from pre-ART plasma, viruses induced from QVOA cultures, or ART-interruption are shown with a horizontal black arrow. Clades with bootstrap values >70 are shown. Abbreviations: ARV: antiretroviral; AZT: zidovudine; DDI: didanosine; EFV: efavirenz; FTC: emtricitabine; 3TC: lamivudine; IDV: indinavir; NNRTI: non-nucleoside reverse transcriptase inhibitor; NRTI: nucleoside reverse transcriptase inhibitor; RLP: rilpivirine; D4T: stavudine; TAF: tenofovir alafenamide; TDF: tenofovir disoproxil.

RV were genetically characterized by sequencing the C2V5 region of HIV *env* from plasma diluted to single viral templates (i.e., single genome sequencing, **SGS**) [37]. Three participants were studied from the time of acute HIV infection over a median of 13.2 years (**Figs 1–3**). Participants 83747 (hereafter referred to as “1”), 59530 (“2”), and 50877 (“3”), from the Seattle Primary Infection Cohort met inclusion criteria for this study: 1) ART initiation after ~3 years of infection; 2) suppression of plasma HIV RNA to <50 copies/mL for ≥5 years; 3) a period of ART-interruption with documented rebound viremia with study specimens available; and 4) re-initiation of ART following virus rebound (**S1 Fig**). ART was first initiated after a median of 3.6 years of “chronic” infection (**Table 1**). ART-suppression (VL<50c/mL) in Participants 1–3 spanned 8, 7.6, and 6.8 years prior to self-initiated ART-interruptions of 76 days, 20–31 days, and 3 years, respectively.

**Table 1:** Participants’ characteristics.

Participant	Sex	Age at HIV diagnosis	Fiebig Stage at study enrollment	Time to ART-initiation (years)	CD4 T-cells at ART initiation (cells/uL)	Total years studied
**1**	M	31	I	4.0	340	13.2
**2**	M	46	I/II	2.9	287	13.0
**3**	M	33	IV	3.6	643	13.7

Plasma specimens with volumes ranging of 3–21 mL were used for SGS, including 3–5 “pre-ART” timepoints, 3–4 during “ART-suppression#1” timepoints (i.e., plasma HIV RNA <50c/mL), 1–3 “ART-interruption” timepoints (i.e., plasma HIV RNA >50c/mL following ART-interruption and shortly after re-initiation of ART), and 1–2 “ART-suppression#2/#3” timepoints (i.e., plasma HIV RNA <50c/mL); **Fig 1A, Fig 2A, Fig 3A** show when specimens were collected and analyzed. Negatively selected CD4^+^ T cells from specimens collected 6.6, 7.3, and 3.1 years following ART-initiation from Participants 1–3, respectively, were used in a 28-day QVOA (format shown in **S2 Fig**), with C2V5*env* SGS derived from supernatants of p24-antigen (+) wells. The total number of SGS are shown in **Table 2**. Plasma collected during ART-suppression yielded a mean of 3.3, 1.8, 1.5 SGS/mL from Participants 1–3, respectively (**S3 Fig**). RV levels were relatively consistent within each participant over time, except for two outlier timepoints when high frequencies of identical sequences were detected (Participant 2 at 7.3 years and Participant 3 at 6.3 years of infection when 90% (36/40) and 89% (8/9) of sequences were identical, respectively). Laboratory quality control included maximum likelihood phylogenetic analyses of all sequences generated in this project (**S4 Fig**) and within our laboratory; and these found a lack of cross-contamination between the 3 participants’ specimens or with viral sequences from other projects.

**Table 2:** Number (%) of HIV C2V5*env* SGA sequences and genetic variants derived from participants’ plasma and QVOA by antiretroviral status.

Time	Source	Variable	Participant		
			1	2	3
**Pre-ART (VL: 923–1.4 million c/mL)**	**Plasma**	Total # sequences	123	64	80
		# unique sequence variants	87	47	74
		# (%) sequences from putative clones	36 (29%)	17 (27%)	6 (8%)
		# (%) unique X4-tropic sequence variants	0	1 (2%)	0
**RV, ART-suppression #1 (VL<50c/mL)**	**Plasma**	Total # sequences	131	74	44
		# unique sequence variants	75	25	26
		# (%) sequences from putative clones	56 (43%)	49 (66%)	18 (41%)
		# (%) unique X4-tropic sequence variants	41 (55%)	0	0
	**QVOA**	Total # sequences	64	158	64
		# unique sequence variants	28	27	15
		# (%) unique X4-tropic sequence variants	10 (36%)	0	0
	**Infectious viruses linked by sequence identity**	# (%) unique RV variants linked to pre-ART	6 (8%)	1 (3%)	1 (3%)
		# (%) unique RV variants linked to QVOA	5 (6%)	0	1 (3%)
		# (%) unique RV variants linked to rebound	2 (3%)	2 (6%)	3 (8%)
**ART-interruption (VL: 73–268,940c/mL)**	**Plasma**	Total # sequences	52	43	62
		# unique sequence variants	41	7	38
		# (%) sequences from putative clones	11 (21%)	36 (84%)	24 (38%)
		# (%) unique X4-tropic sequence variants	7 (17%)	0	7 (18%)
**RV, ART-suppression #2/3 (VL<50c/mL)**	**Plasma**	Total # sequences	23	22	15
		# unique sequence variants	5	10	13
		# (%) sequences from putative clones	18 (78%)	12 (55%)	2 (13%)
		# (%) unique X4-tropic sequence variants	4 (80%)	0	3 (23%)

**Fig 4:**
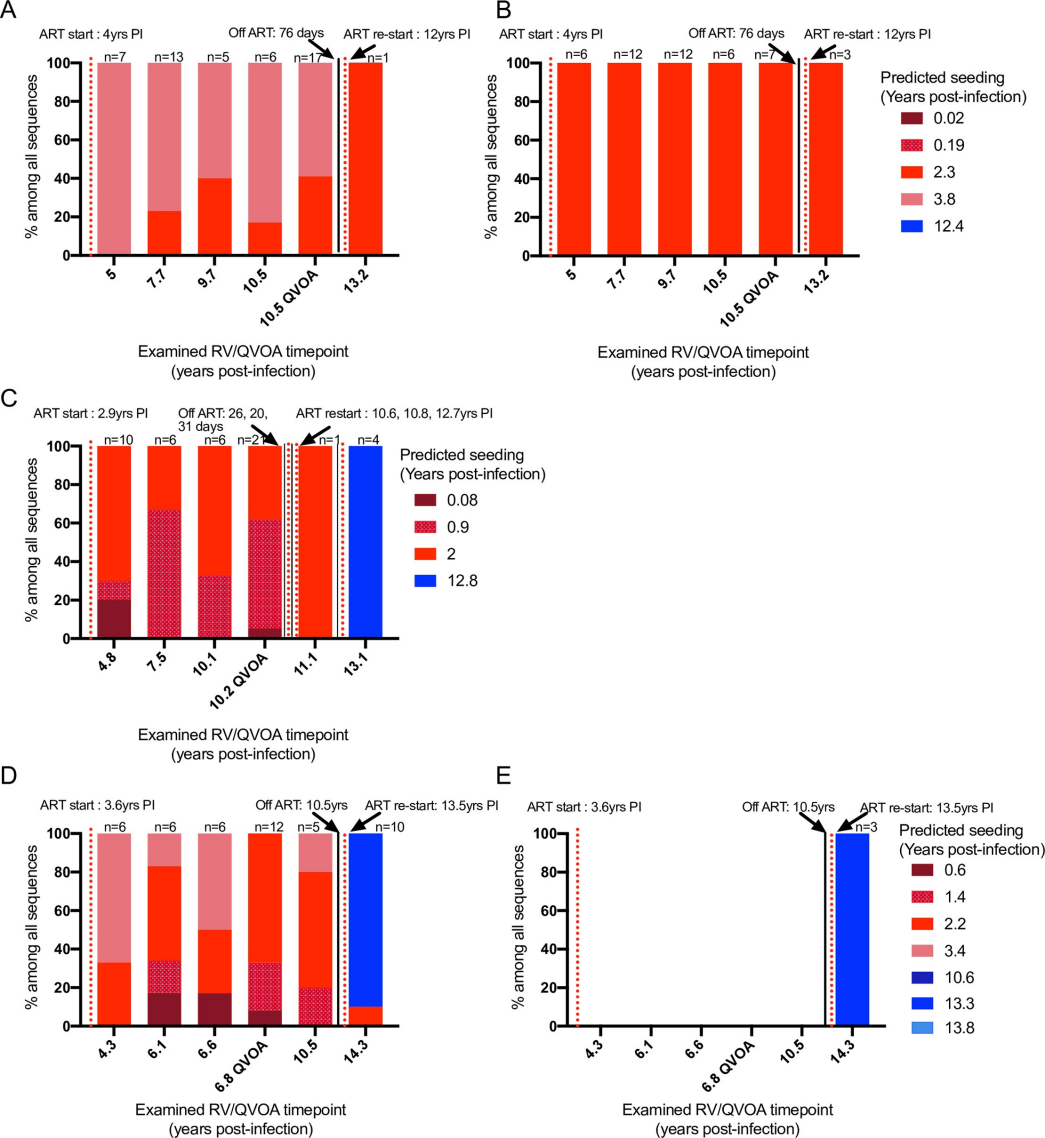
Pairwise distances reveal that RV and QVOA-producing proviruses were seeded across the pre-ART timeframe. The proportion of RV/QVOA sequences at each timepoint predicted to have been seeded at a specific pre-ART or ART-interruption timeframe are shown for Participant 1 (**A,B**), Participant 2 (**C**), and Participant 3 (**D,E**). Seeding of imputed unique R5- (**A, C, D**) and dual and X4-tropic RV (**B** and **E**) are shown separately. The time of pre-ART (red gradient colors) and ART-interruption (blue gradient colors) that HIV replication is predicted to have seeded the reservoir that persisted during ART-suppression and produced each unique RV or QVOA-derived virus is shown with different gradient colors. A total of 4, 3, 4 pre-ART and 1, 1, 3 ART-interruption timepoints were used to infer reservoir seeding time for the RV/QVOA-producing proviruses in Participants 1–3, respectively. ART-interruption timepoints for Participants 1 and 2 included 1 timepoint after ART re-initiation when viral levels were above the limit-of-quantification of our assay (50c/mL). Red dotted line indicates the timepoint ART was initiated. Black solid line indicates the timepoint ART was interrupted. Total (n) number of unique R5 and X4-tropic RV and QVOA-derived sequences plotted are shown above the bar.

In phylogenetic analyses, RV and QVOA sequences clustered with multiple pre-ART clades from each participant (**Figs 1B, 2B and 3B**). To better understand the timeframe when the RV- and QVOA-producing cells “seeded” the HIV reservoir that persists during ART (i.e., when clones were established), pairwise distances were compared between: 1) RV/QVOA sequences from ART-suppression#1 and pre-ART sequences; and 2) between RV sequences from ART-suppression#2/#3 and pre-ART and ART-interruption plasma sequences. The shortest pairwise distance to each unique RV/QVOA sequence was predicted to be the time the clone was established. Cell populations contributing to the RV throughout ART-suppression#1 were estimated to have seeded the reservoir in large part just prior to ART-initiation for all 3 participants (**Fig 4**), although seeding occurred as early as 28 days following the estimated date of acute infection for Participant 2 (**Fig 4C**). After a year of ART, a greater proportion of RV were from clones established at earlier timepoints in Participants 2 and 3 (**Fig 4C and 4D**). RV from ART-suppression#2/#3 included clones seeded prior to ART-initiation in Participants 1 and 3, and included viruses predicted to use CCR5 and CXCR4 as co-receptors (**Fig 4A, 4B, 4D and 4E**). However, during ART-suppressions#2/#3, RV were composed primarily of variants that evolved during ART-interruptions (**Fig 1B, Fig 2B, Fig 3B**), including 100% of RV variants from ART-suppression#3 in Participant 2 and 90% of the R5-tropic RV variants from ART-suppression#2 in Participant 3 (**Fig 4C and 4D**).

**Fig 5:**
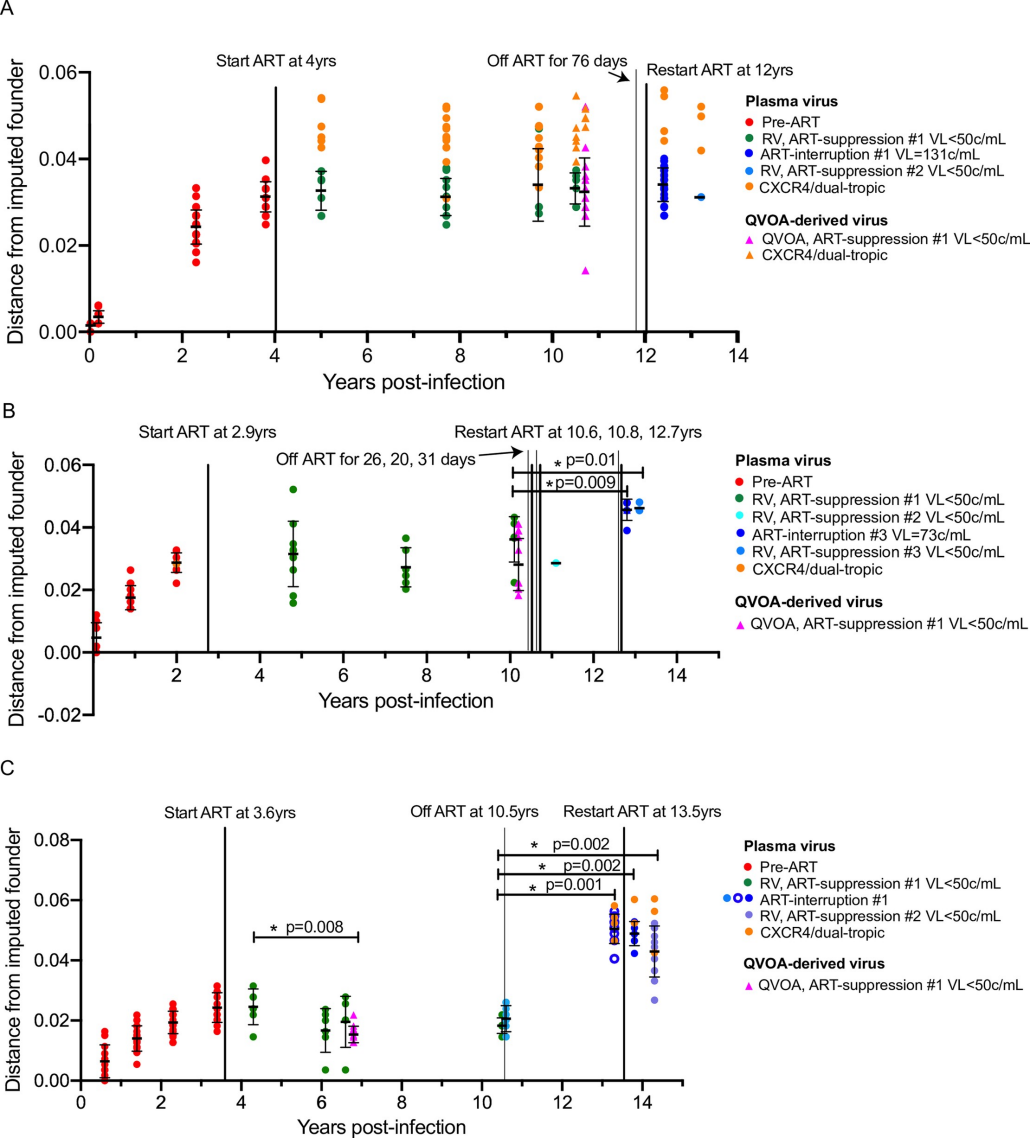
Divergence of viral populations from the imputed founder sequence through time. Each panel shows pairwise distances (y-axis) of unique plasma and QVOA-derived sequences from the imputed founder (means ±SD indicated for each timepoint with black horizontal lines) for Participant 1 (**A**), 2 (**B**), and 3 (**C**) over time (x-axis). Treatment status or imputed sequence tropism is indicated by color, shown in key. The times ART was initiated or suspended are shown with thick and thin black vertical lines, respectively. ART was suspended for 76 days, 26–31 days (on 3 separate occasions), and 3 years in Participants 1–3, respectively. Statistically significant differences (*p<0.05) in divergence of the RV/QVOA-derived viruses relative to the first RV timepoint as determined by 2-sample Wilcoxon rank-sum test are indicated. Also indicated are significant differences between the ART-interruption and RV after re-suppression relative to the latest RV timepoint during ART-suppression #1.

The genetic diversification (**S5 Fig**) and divergence (**Fig 5**) of plasma variants from the founder sequences of the 3 participants prior to ART-initiation revealed the expected time-ordered increase in genetic distance and diversity. During ART, analyses of all RV sequences suggested continued divergence in Participant 1 (**Fig 5A and S5A Fig**); however, this was due to the detection of X4 variants, which were not detected prior to ART-initiation. The R5 RV variants across the 3 participants did not diverge (**Fig 5**). Divergence of plasma sequences resumed upon ART-interruption in Participants 2 and 3 (**Fig 5B and 5C**).

**Fig 6:**
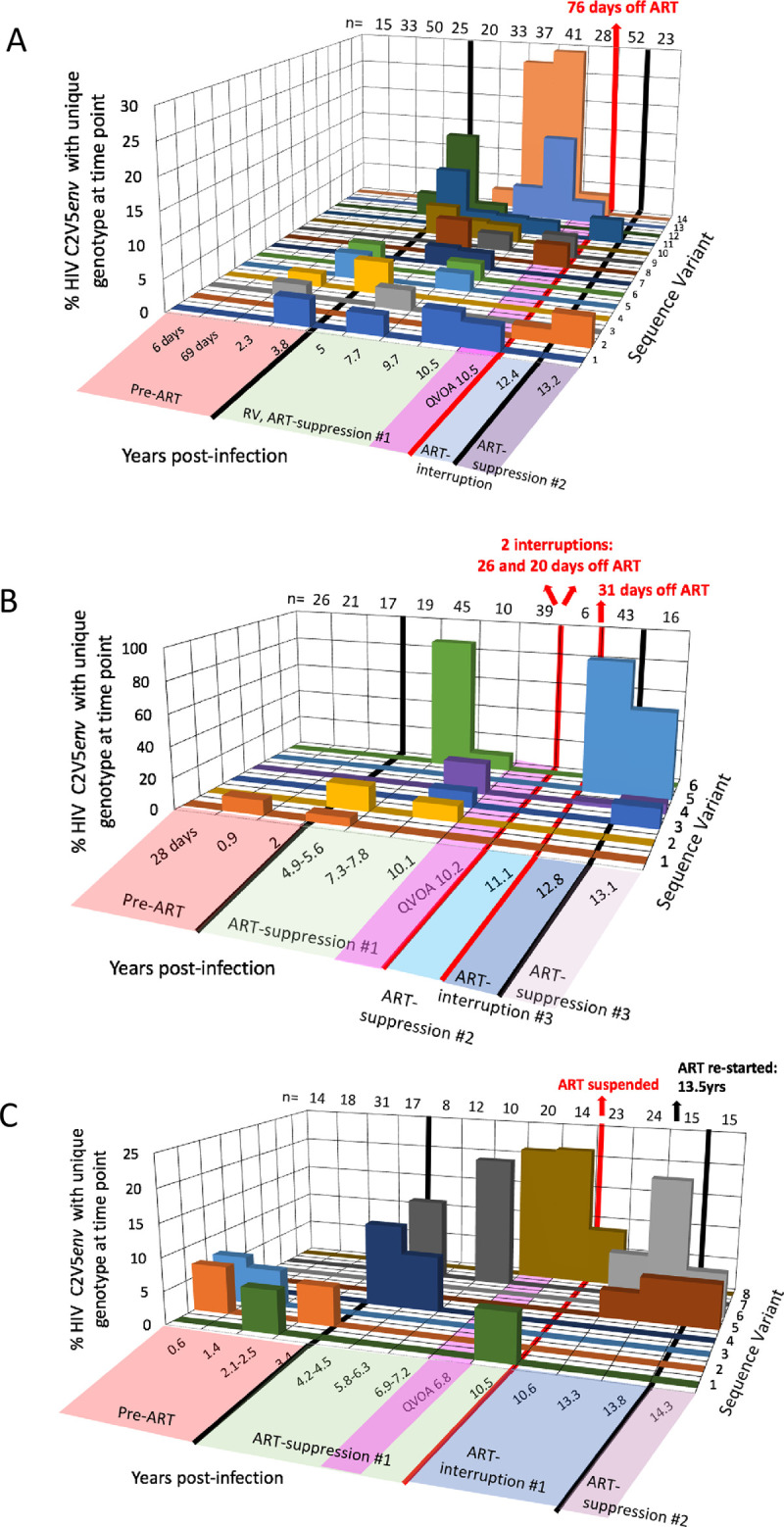
Persistence of identical HIV C2V5*env* variants over time. Each panel shows data for one study participant (Participant 1 (**A**), 2 (**B**), and 3 (**C**)). Persisting unique plasma and QVOA-derived variants detected at ≥2 plasma timepoints, and thus imputed to be from a clonal cell population, are shown by different colors (z-axis) with proportion (y-axis) of the variant among all sequences obtained at that timepoint shown. Multiple identical plasma variants detected at only one timepoint are not graphed. Time since the estimated date of HIV infection and ART status is indicated along bottom of x-axis. ART-interruptions are noted by red lines transecting z- and y-axes with durations noted above red arrows. Numbers along the top of each panel represent the total number of sequences derived during each timepoint.

Cell clones imputed to harbor infectious HIV have been documented to persist for up to 5 years [13, 14, 17, 22]. In our study participants, approximately half of the RV variants detected during ART-suppression#1 may have been derived from clones by virtue of identical C2V5 sequences, including 43%, 66%, and 41% from Participants 1–3, respectively (**S6 Fig**). RV with identical C2V5*env* sequences detected at ≥2 timepoints, and thus likely from infected clones, included 14/34 (41%), 6/13 (46%), and 8/28 (29%) variants with identical sequences observed in RV over a median of 3.9 years (range: 0.8–7.4), 3 years (range: 3–3.9), and 1 year (range: 0.1–8.2), in Participants 1–3, respectively (**Fig 6**). The persistence of identical RV variants was not associated with imputed cell tropism.

The prevalence of each unique RV sequence variant fluctuated significantly over time in Participants 1 and 2 (p<0.0001, 2xC Fisher’s exact test), demonstrating dynamic shifts in the cell populations producing RV. However, genetic heterogeneity of RV sequences was not found in Participant 3 (p = 0.131) likely due to our derivation of fewer RV sequences. The RV variants that persisted over years were detected as multiple populations of minority frequency variants in the three participants. Two RV variants were detected as majority frequency variants (>50%) at single timepoints across the 9 timepoints evaluated (at 7.3–7.8 and 13.1 years) in Participant 2 (**Fig 6B**).

RV sequences were identical to multiple sequences amplified from pre-ART plasma, QVOA, and ART-interruption “rebound” plasma, including 8/154 (5%), 6/154 (4%), 7/154 (5%) from Participants 1–3, respectively (**Table 2**). The majority of these potentially infectious RV variants (20/21; 95%) were detected in ≥2 plasma specimens, indicating that they likely came from infected clones (the exception is a RV sequence detected at one timepoint that was identical to QVOA-derived virus, Variant 9 from Participant 1 detected at 9.7 years post-infection (**Fig 6A**)). Identical sequences were observed between seven RV or QVOA variants and “rebound” ART-interruption plasma variants (Participant 1, Variant 2 and 11; Participant 2, Variants 4 and 5; and Participant 3, Variants 5, 6, and 8) (**Fig 6**), suggesting rebound from infected cell clones. These included variants detected across long timespans: 7.4 years for Participant 1’s Variant 11 and 2.7 years for Participant 2’s Variant 4 and 3.8 years for Participant 3’s Variant 8 (**Fig 6**).

To assess whether RV or rebound C2V5*env* sequences were glycosylated, which could promote escape from neutralizing antibodies and contribute to persistence [38, 39], the number of potential N-linked glycosylation sites (**PNLGS**) in each sequence were tallied and compared over time. PNLGS across RV sequences were increased compared to ART-interruption sequences in Participants 1 and 3 (**S7 Fig**). However, consistent patterns in PNLGS distributions were not detected across the three participants.

## Discussion

Residual viremias provide a window into the segment of the HIV reservoir that actively produces virions during ART-suppression. Our longitudinal analyses of RV sequences reveal that this “active” HIV reservoir is dynamic, and that a subset of RV are genetically linked to ART-suppression viruses induced in QVOA and/or ART-interruption viruses in “rebound” plasma; hence to the infectious HIV reservoir. These findings indicate that, in addition to intact proviruses in the latent reservoir, the cells that produce RV appear to be a critical target for cure strategies.

Genetic distances and sequence identity between RV, QVOA, and rebound viruses have been examined in multiple studies to gain insights into the infectious reservoir. We found relatively few identical variants between RV and QVOA and few between RV and rebound viruses, which aligns with others’ findings [30, 35, 40] and [22], respectively. While our detection of RV that shared identical sequences with replicating viruses detected in QVOA and rebound viruses were not common, these observations support the hypothesis that the infectious reservoir that contributes to rebound viremia also produces RV. We detected identical sequence variants, albeit relatively few, between QVOA-derived and rebound viruses; similarly, we observed that many QVOA and rebound viral sequences intermingled in phylogenetic analyses, suggesting shared genetic lineages. Studies by others [41] have sought but not found sequence linkages between these virus populations, with one study reporting that viral outgrowth assays (**VOA**) and rebound viruses clustered separately in phylogenetic analyses of twelve individuals’ sequences [41]. The linkages between QVOA-derived and rebound viruses in our study may reflect greater sampling of an extensive viral reservoir compared to the latter study. Overall, our study appears to provide novel evidence that clonal cell populations within the persisting HIV reservoir produce RV and infectious variants that contribute to rebound viremia upon ART-interruption.

The HIV-infected cell reservoir producing RV and viruses detected in QVOA in our participants appeared to have been seeded throughout the pre-ART period. The genetic distances between RV and pre-ART viruses appeared to transition across the time of ART-suppression, progressing from RV genetically similar to variants just prior to ART-initiation to variants from across the pre-ART period. This transition is consistent with the idea that the majority of RV detected immediately after ART-initiation come from recently infected cells; however, following the expected decay of these cells [33, 42–45], a greater proportion of RV is then detected from long-lived cell populations. Others’ examinations of the time for reservoir seeding include a recent study of viral sequences that predicted seeding of the infectious reservoir occurs across the pre-ART period [46]; similar to findings in our participants. Another group observed seeding of the majority of on-ART QVOA-derived viruses 1 year prior to ART-initiation [42]. This latter pattern suggests that the population of cells replicating immediately prior to ART did not decay rapidly, and suggests that in the individuals studied that this population of infected cells was perhaps renewed due to imperfect adherence to ART. Our observation that following ART-interruption and re-suppression (i.e., during ART-suppression-#2/3), the RV included new variants not detected during the ART-suppression-#1, provides data in support of the hypothesis that recent HIV replication partially resets and/or obscures the previously seeded long-lived reservoir.

The prevalence of each RV variant fluctuated across ART-suppression. Identical RV sequence variants observed to persist over time, were detected over a median of 3.9 years, including after ART-interruption and during ART-re-suppression. The recurrent detection of identical RV sequences at multiple timepoints is consistent with the production of RV by clonal cell populations; as one cell would be unlikely to persist over this timespan with repeated production of virions at a burst size [36] required to produce RV. Our finding that RV sequence variants fluctuate over time confirm those of others’ studies [14, 30, 40, 44]. This variation in the representation of RV variants may be due to diverse antigens periodically stimulating different clonal cell populations that then produce virions [14], differential CTL immune recognition and elimination of RV-producing clones [24, 47, 48], and/or a gain or loss of gene function due to the location of the HIV integration site [49]. The specific clone producing persistent viremia during ART was previously identified by viral sequence and HIV integration site in an individual with cancer, with the provirus integrated in a repetitive element [22], which impedes the study of virally modulated gene expression. Given that we detected multiple RV variants that persisted over years, we hypothesize that clones producing RV are selected either for escape from CTL recognition or dysfunctions in immune elimination; both paths to “immune evasion” imply that RV-producing clones represent a particularly challenging but critically important reservoir to target for cure strategies.

The contribution of RV variants to rebound viremia was not associated with whether the clone was detected frequently or rarely among RV sequences. This suggest that multiple or disparate factors likely contribute to RV production and viral rebound, or that our sampling was too sparse to detect weak associations.

While previous studies of low-level intermittent viremias (plasma HIV RNA 50–400 c/mL) [29, 50] occasionally detected evidence of HIV replication, genetic divergence of our participants’ sequences was detected only after frequent or prolonged ART-interruptions. No significant increase in viral divergence was observed among plasma sequences imputed to use the CCR5 co-receptor during the first period of ART suppression in all 3 participants, suggesting the lack of ongoing viral replication during effective ART. However, the absence of detectable divergence despite viremia during ART-interruptions of Participants 1 and 2 demonstrates that divergence is insensitive to short periods of low-level virus replication, and emphasizes that a lack of divergence does not provide certainty that HIV replication did not contribute to RV. The detection of multiple RV sequences predicted to use the X4 co-receptor that cluster closely in the phylogenetic analyses of Participant 1, and in multiple individuals in previous studies of low-level viremias [29, 50] leads us to speculate that X4 tropism is selected for persistence and/or replication during ART-suppression.

The increase in PNLGS in the RV of two participants provide a weak suggestion that escape from antibodies may select HIV variants that persisted and were expressed during ART. However, the significance of this observation is uncertain given our small study population and the absence of a pattern across the three participants.

Given the extremely low levels of viremia under study, only a limited, albeit highly variable region of the viral genome (the ~600bp C2V5 region of *env*) was assessed, which may have overestimated “identical” populations due to sequence differences outside of this region. To increase the power of this analysis, we studied individuals likely to have substantial viral diversity when ART was initiated, i.e., approximately three or more years of untreated infection. The Clonal Prediction Score Calculator [51] (https://silicianolab.johnshopkins.edu/cps/#/) estimated that identical sequences over the examined region of the viral genome provided an 83% certainty that the sequences were identical throughout the genome of persons who initiated ART during chronic infection. Our QVOA studies only sampled infectious viruses induced from PBMC. Examination of other tissues where replication is hypothesized to occur, such as lymphatic tissues [52], may have provided further insight as to the viral variants contributing to rebound following ART-interruption.

Our findings suggest that despite years of effective ART, certain cell clones expanded by proliferation are selected to persist and some of these produce RV, even after ART-interruption. The persistence of these virus-producing clones for at least 8.2 years during effective ART implies that immune elimination of infected cells may be hampered due to CTL-escape mutations or dysfunctional immune responses. Multi-faceted strategies that use immunotherapies may be needed to cure HIV infection. These would need to include strategies to target proviruses that escape CTL via different mechanisms, including latent proviruses integrated in “silenced” regions of the human genome, and latency-reversed or RV-producing cells not effectively targeted by host CTL responses.

## Materials and methods

### Study participants

This study was a retrospective observational study based on prospectively-followed participants from the Seattle Primary Infection Clinic (PIC) cohort who were enrolled from 1992–2015 [53–56]. Participants were selected based on the following inclusion criteria: 1) initiated ART during chronic infection; 2) ART-suppressed for ≥5 years with all plasma HIV RNA loads <50c/mL; 3) multiple plasma specimens available prior to and during ART-suppression; and 4) a treatment interruption with plasma specimens available following re-initiation of treatment. At the time of cohort entry, all participants had either acute or early HIV infection [56]. ART was initiated based on the availability of potent antiretroviral therapy, the strength of consensus treatment recommendations, and personal preferences. Self-initiated treatment interruptions for the 3 participants included in our study were for reasons including insurance lapse and a decision made in conjunction with a clinical care provider.

### Ethics statement

The University of Washington Institutional Review Board approved this study, and all participants provided written consent. Participants initiated treatment under the care of their clinicians as recommended by guidelines and personal preferences.

### Study schema

Participants’ study visits and specimen collections were as frequent as weekly for the month following enrollment, monthly for three months, at eight-week intervals through the remaining first year of observation, and then at a minimum of six-month intervals, although the frequency varied somewhat within the cohort. Multiple longitudinal plasma specimens were used to derive viral RNA and 1 timepoint during ART suppression (6.5, 7.25, 3.08 year after ART initiation), where leukapheresis specimens were available, was used for the QVOA for Participants 1–3, respectively.

### QVOA

Total CD4^+^ T cells (~10^7^) from 1 timepoint during ART suppression were purified by negative selection (Miltenyi, Catalog # 130-096-533) from participant PBMC, serially diluted (3-5-fold) into 48-well plates (2–8 replicates/CD4^+^ input) containing monocytes (10^5^/well) purified from a healthy HIV-seronegative donor. Cocultures were maintained for 3 days in 1mL culture media (Iscoves with 10% FCS) containing 1ug/mL anti-CD3 OKT3 (Miltenyi, Catalog # 130-093-387). At days 3 and 10 of coculture, the supernatant was removed, and CD8-depleted allogeneic PHA-blasts from a healthy HIV-seronegative donor (10^5^/well) were added to the cocultures in media containing IL-2 (PeproTek, Catalog # 200–02). Cocultures were maintained for 28 days and supernatants harvested weekly for virus outgrowth by HIV p24 antigen. The infectious units per million CD4^+^ T cells (IUPM) was calculated using the maximum likelihood estimate on days 15, 21, and 28 of culture [57].

### Plasma RNA extraction

RNA was extracted from 1-21mL aliquots of participants’ plasma or 50-140uL of QVOA supernatants. When plasma contained HIV RNA at <50c/mL, virus particles were pelleted by centrifugation at 14,000rpm for 90 min at 4°C, with removal of all but ~140uL of plasma over the pellet. The Qiagen QIAmp Viral RNA Mini kit (Qiagen, Catalog # 52906) was then used as directed by the manufacturer to extract the RNA. When viruses were pelleted, lysis buffer was added directly to the pellet and remaining plasma and once resuspended, was transferred to a new 1.5mL Eppendorf tube for the extraction, followed by ~500uL of PBS used to wash any remaining virions from the tube. A positive control plasma sample containing >100,000 copies/mL of HIV RNA was similarly processed with each extraction. RNA was eluted into 60uL of AVE buffer (Qiagen) and immediately DNase-treated to degrade contaminating HIV DNA using Invitrogen DNase I, Amplification Grade (Catalog # 18068–015) following the manufacturer’s instructions (1uL of 10X buffer was added to the eluted RNA followed by 1uL DNase I). The mixture was incubated at room temperature for 15 min, the enzyme was then deactivated with the addition of 1uL 25mM EDTA and a 10 min incubation at 65°, and then put on ice. RNA from the QVOA supernatants were similarly DNase I-treated with the exception of 6/28 (21.4%) QVOA p24Ag(+) wells from all 3 participants when DNase I was not available. RNA was immediately converted to cDNA (see below).

### cDNA synthesis

All 60uL of DNase-treated RNA was converted into cDNA using the Invitrogen Superscript IV (SSIV) enzyme kit (Catalog # 18090200). Briefly, to the 60uL RNA, 5.5uL of 10mM dNTP, 5.5ul of each of the 20uM of HIV *pol*-specific forward RTA primer (HXB2 positions 3328–3303) and *env-*specific reverse BH2 primer (HXB2 positions 7697–7725) were added and the mixture incubated in a thermocycler at 65° for 5 min and then immediately put on ice for at least 1min. Subsequently, 2.75uL of molecular grade water, 22uL 5X RT buffer (Invitrogen, catalog # 18090200, 5.5uL 0.1M DTT, 2.75uL Promega RNasin Plus RNAse inhibitor, 10,000U (Catalog # N2615), and 1uL of the SSIV enzyme was added and incubated at 55° for 90min, 80° for 10min, and then held at 12° prior to storing at -20°.

### Endpoint nested PCR of HIV C2V5*env*

Sequences of the C2V5 region of *env* were derived from specimens diluted to single HIV templates [37, 58]. These sequences were derived following limiting dilution (i.e., by single genome amplification, SGA) to minimize recombination, resampling and sequencing errors [59, 60]. The first-round 50uL PCR included: 2-3uL diluted cDNA, molecular grade water,10uL Bioline 5x MyTaq Reaction Buffer (Catalog # BIO-21107), 1ul each of the 20uM forward *env*-specific primer ED31X (HXB2 positions 6865–6831) and reverse *env*-specific primer BH2 (HXB2 positions 7697–7725) and 0.5ul Bioline MyTaq DNA polymerase (5U/ul). The nested second-round 25uL PCR reaction included: 2uL of first-round PCR product, molecular grade water, 5uL 5x MyTaq Reaction Buffer, 0.5uL each of the 20uM forward primer DR7 (HXB2 positions 6990–7021) and reverse primer DR8new (HXB2 positions 7631–7653), and 0.25uL MyTaq DNA polymerase (5U/uL). A PCR positive control (10 copies of 8E5 cellular DNA) provided by NIH AIDS Reagent Program and a negative control (water) were included with each run. The cycling conditions for both rounds of PCR were: 94° for 5min, 34 cycles of 94° for 20sec, 55° for 20sec, 72° for 1min, followed by 1 cycle at 72° for 7min and a hold at 8°C. In some cases, an alternate second-round PCR reverse primer env7656 was used (HXB2 positions 7656–7690), in which case the annealing temperature was 52° instead of 55°.

### DNA sequencing and phylogenetic tree analyses

The ~610 bp C2V5*env* gene amplicons from the 2^nd^ round nested PCR were directly sequenced by the Sanger method using DR7 and DR8new or env7656 primers. All chromatograms were evaluated to ensure the lack of any double peaks and thus confirm single genome sequences (sequences with >1 double peak were eliminated from analyses). The forward and reverse sequences were aligned to a reference HXB2 sequence (Genbank, K03455.1) using Geneious R11.1.5 (https://www.geneious.com) and base calls reviewed for bi-directional consistency. Codon-based DNA sequence alignments were generated in Geneious using the MUSCLE algorithm (version: 3.8.425) [61] and manually refined. Sequences with significant levels of APOBEC-induced G-A hypermutations were determined by Hypermut 2.0, LANL [62]. UniSeq (https://indra.mullins.microbiol.washington.edu/cgi-bin/UniSeq/uniseq.cgi) was used to remove identical sequences from the alignment prior to creating phylogenetic trees in DIVEIN [63] (https://indra.mullins.microbiol.washington.edu/DIVEIN/diver.html) using the GTR substitution model, and best of NNI and SPR tree improvement, and bootstrapping. MEGA version 7 [64] was used to format the output phylogenetic tree from DIVEIN.

### Viral population diversity and divergence from the imputed founder virus

DIVEIN [63] was used to estimate pairwise distances and divergence of unique sequences relative to the imputed founder. C2V5*env* sequences that were significantly hypermutated by Hypermut 2.0, LANL or contained stop codons were removed from analysis. Mean and standard deviations were determined using GraphPad Prism v6.07. Two-sample Wilcoxon rank sum test was performed to evaluate significant changes in RV divergence comparing the first ART-suppression timepoint to each subsequent RV timepoint. Two-sample Wilcoxon rank sum test was also performed comparing sequences at the latest RV timepoint during ART-suppression #1 relative to all subsequent timepoints.

### Reservoir “seeding”

For each unique ART-suppressed sequence, the distance between that sequence and each pre-ART and ART-interruption sequence from the same participant was examined, using pairwise distances obtained from the divergence analysis above. The estimated time of seeding of the provirus contributing to the RV was estimated as the pre-ART or ART-interruption timepoint with the smallest pairwise distance relative to the RV. In case of a tie, the seeding time was estimated to be the earliest time at which we observed pre-ART sequences tied for the smallest distance.

### Cellular tropism

To predict viral co-receptor use, or “tropism”, the plasma and QVOA viral sequences were codon-aligned relative to the V1-V3 region (7,110–7,218bp) of HXB2 (Genbank, K03455.1). Any insertions found in the alignment of plasma virus sequences and sequences of viruses induced from QVOA that were not found in HXB2, were removed from tropism analysis performed using PSSM [65]. Sequences with a percentile >95 or that contained stop codons were excluded from tropism analysis. Tropism predictions are reported as previously described [65]; scores ≤ -7 as CCR5-tropic, -7 to -3 as dual-tropic, and ≥ -3 as CXCR4-tropic.

### N-linked glycosylation

Unique C2V5*env* nucleotide sequence alignments were input into the N-glycosite program [66] and evaluated using the default settings. Output files were used to create the bar graphs in S7 Fig. Sequences that were significantly hypermutated or contained stop codons were removed from alignments.

### Statistical analyses

Fisher's exact test was used to determine whether the distribution of observed variants during ART suppression was homogeneous over time. For each participant, we determined the most common variant (MCV) by pooling variant counts during the ART suppression period. In the case of a tie, the MCV was the variant with the greatest number of counts during the entire study period. Each participant thus contributed a 2xC contingency table, where C is the number of timepoints (non-ordered) during ART suppression for that participant, and observations at each timepoint were dichotomized as being MCV or non-MCV.

To determine the degree to which viruses from pre-ART contributed to residual viremia during ART suppression and re-suppression, we determined the proportion of sequences seen during ART suppression or re-suppression that were also seen pre-ART for each participant. We calculated 95% exact binomial confidence intervals for each proportion reported.

## Supporting information

S1 Fig Study schema. Overview of the selection criteria of the 3 examined participants in the PIC cohort and the timepoints/specimens analyzed.
(TIF)

S2 Fig Schema of quantitative viral outgrowth assay (QVOA) workflow and results for each participant. Panels show QVOA results for each Participants 1 (Panel **A**), 2 (**B**), and 3 (**C**). Cultures utilized participant-derived total negatively-selected CD4^+^ T-cells that, following serial dilution, were co-cultured with monocytes from a healthy, HIV-seronegative donor, activated with anti-CD3 and maintained with IL-2 in culture for up to 28 days. HIV wells yielding virus were detected by testing supernatants for p24 antigen (Ag) by ELISA on days 8, 15, 21, and 28. The infectious units per million (IUPM) cells and 95% confidence intervals were calculated at day 28 of culture. Colors indicate semi-quantitative amount of p24Ag detected. Abbreviations: CM: culture media. BF: PHA stimulated CD8-depleted PBMC 72hour Blast Feed.
(TIF)

S3 Fig HIV virion concentrations in plasma. Each panel shows minimal concentration of virions (y-axis) estimated in specimens analyzed from Participants 1 (**A**), 2 (**B**), and 3 (**C**) at indicated timepoints (x-axis) following the estimated date of HIV infection. Each data point represents the total number of C2V5*env* sequences derived by SGA divided by the volume of each plasma aliquot, with means and standard deviations across aliquots from each timepoint shown. The time when ART was initiated or interrupted are shown with dotted and thin black vertical lines, respectively.
(TIF)

S4 Fig Phylogenetic analysis of HIV C2V5*env* gene sequences from the 3 participants. Participant’s 1–3 plasma and QVOA-derived single genome sequences are represented by green, blue and red, respectively, with each dot representing one sequence. A maximum likelihood phylogenetic tree of all 3 participants’ sequences shows segregation and clustering of sequences by participant indicating the lack of cross-contamination or sample mislabeling.
(TIF)

S5 Fig Diversity of unique C2V5*env* variants from plasma and QVOA-derived viruses over time. HIV-1 pairwise diversity within each specimen is shown with median and range of longitudinal unique plasma and QVOA-derived sequences for Participant 1 (**A**), Participant 2 (**B**), and Participant 3 (**C**). Colors represent comparisons between sequences from CCR5 vs. CCR5 (blue), CCR5 vs. dual/X4 (green), and dual/X4 vs. dual/X4 (magenta) tropisms. QVOA-derived sequences are indicated. The time when ART was initiated or interrupted are shown with dotted and thin black vertical lines, respectively.
(TIF)

S6 Fig Proportions of identical and unique HIV C2V5*env* single genome sequences amplified from plasma. Each panel shows data for one participant: Participant 1 (**A**); Participant 2 (**B**); and Participant 3 (**C**). Each “donut” within the panels represents a timepoint with the segment colors representing identical variants at 1 or >1 timepoint. Shown is the proportion of HIV C2V5*env* sequences with each unique variant among all sequences amplified from that timepoint, with the total number of viral templates sequenced noted in the center of each donut. All variants detected only once are grouped in the white section of the donut. The time since the estimated date of HIV infection is indicated above the donut, and plasma HIV RNA load, if detectable, is noted either below the donut or to the left side of each panel. Variants detected in ≥2 specimens are shown with the same color. The donuts are organized horizontally within each panel by antiretroviral status, as described to the left of each panel. Numbers corresponding to each color in the key represent the specific variant with the same number and color maintained for persistent variants detected over time.
(TIF)

S7 Fig Predicted N-linked glycosylation sites (PNLGS) in unique plasma and QVOA C2V5*env* variants. “N-glycosite” at Los Alamos National Laboratory (LANL) website (URL: https://www.hiv.lanl.gov/content/sequence/GLYCOSITE/glycosite.html; accessed on 9-12-19) was used to determine the fraction of N-linked glycosylation sites among all unique plasma and QVOA-derived C2V5*env* sequences from Participants 1 (**A, D**), 2 (**B, E**), and 3 (**C, F**). Panels A-C show the fraction of N-linked glycosylation sites (Y-axis) among unique sequences at positions mapped to the HIV reference genomes HXB2 (Genbank, K03455.1). Colors and symbols represent different plasma and QVOA timepoints. Dotted lines represent known HXB2 N-linked glycosylation sites (n = 12 sites) [67]. Red and green boxes represent the V3 and V4 regions in the HXB2 C2V5env sequence, respectively. Only unique C2V5*env* gene sequences that did not contain stop codons and that were not significantly G-A hypermutated are shown. Panels D-F show the mean +/- standard deviation of N-linked glycosylation sites among all unique plasma and QVOA sequences. The number of unique sequences included in the analysis is shown in the key. *p<0.05, 2-sample Wilcoxon rank sum test, adjusted for multiple comparisons using the Holm method, was performed to compare levels of glycosylation among RV during ART-suppression #1 relative to sequences from all other timeframes.
(TIF)
