# Supplementary figures and images for: Cells producing residual viremia during antiretroviral treatment appear to contribute to rebound viremia following interruption of treatment
Source: PLoS Pathog. 2020 Aug 25;16(8):e1008791. doi: 10.1371/journal.ppat.1008791 (PMC7473585; doi:10.1371/journal.ppat.1008791)

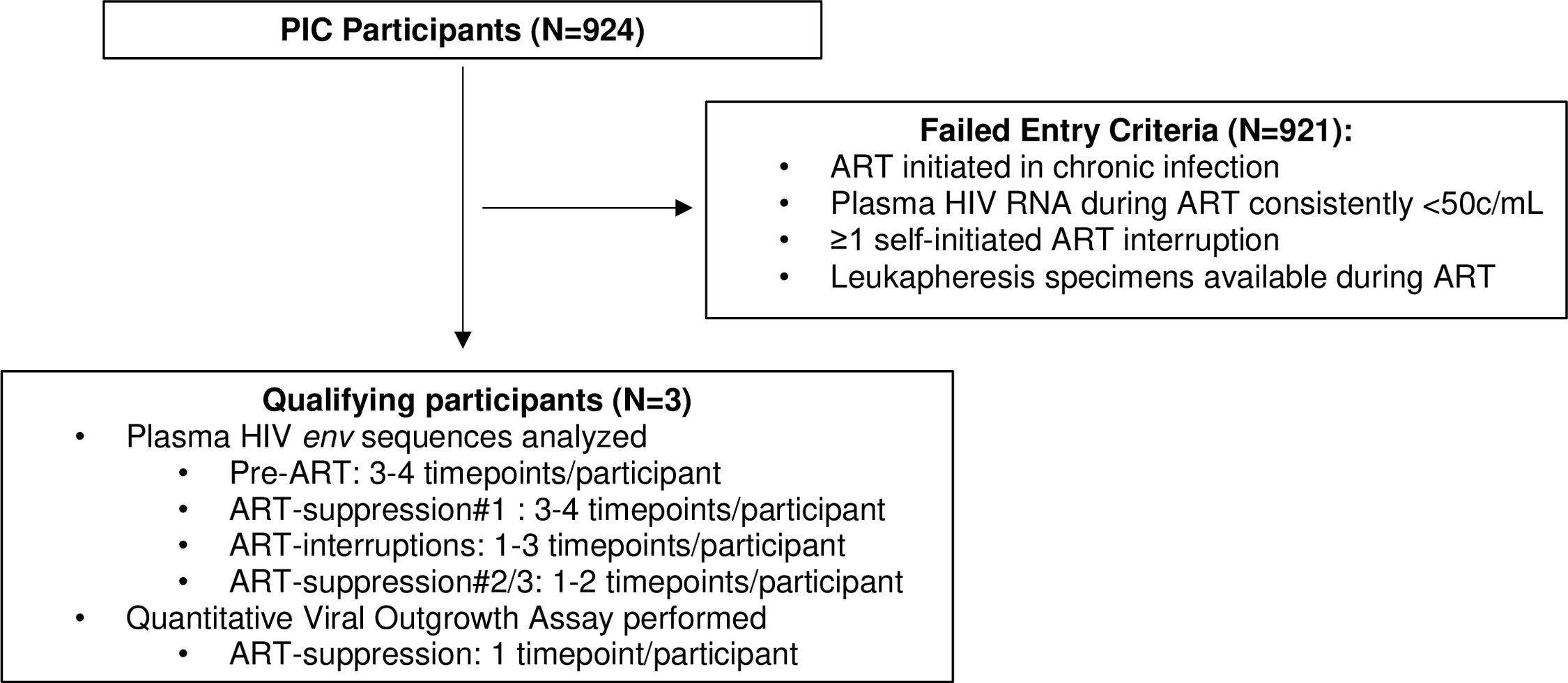

Supplement: S1 Fig — Overview of the selection criteria of the 3 examined participants in the PIC cohort and the timepoints/specimens analyzed. (TIF) [file ppat.1008791.s001.tif]

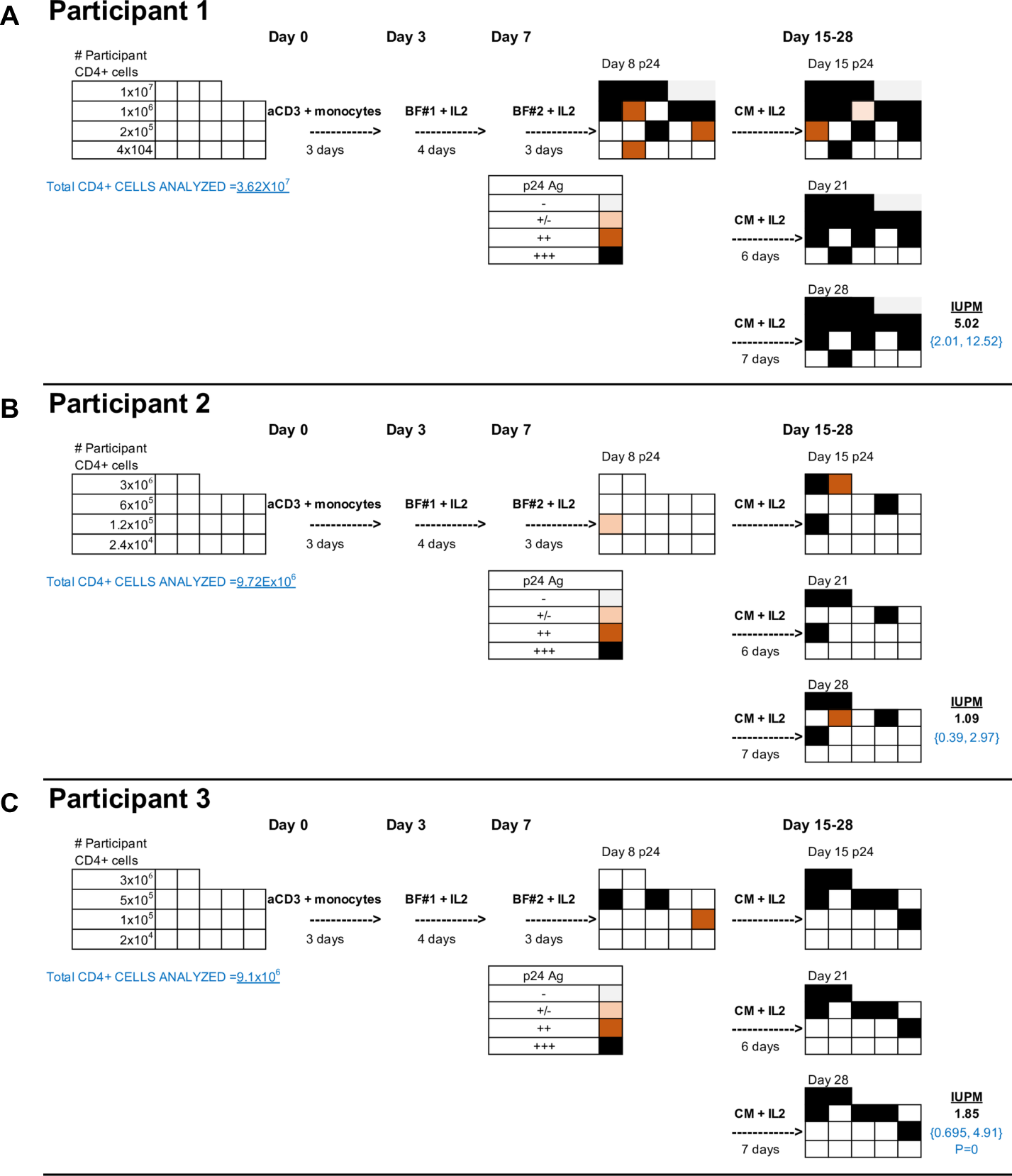

Supplement: S2 Fig — Panels show QVOA results for each Participants 1 (Panel A), 2 (B), and 3 (C). Cultures utilized participant-derived total negatively-selected CD4+ T-cells that, following serial dilution, were co-cultured with monocytes from a healthy, HIV-seronegative donor, activated with anti-CD3 and maintained with IL-2 in culture for up to 28 days. HIV wells yielding virus were detected by testing supernatants for p24 antigen (Ag) by ELISA on days 8, 15, 21, and 28. The infectious units per million (IUPM) cells and 95% confidence intervals were calculated at day 28 of culture. Colors indicate semi-quantitative amount of p24Ag detected. Abbreviations: CM: culture media. BF: PHA stimulated CD8-depleted PBMC 72hour Blast Feed. (TIF) [file ppat.1008791.s002.tif]

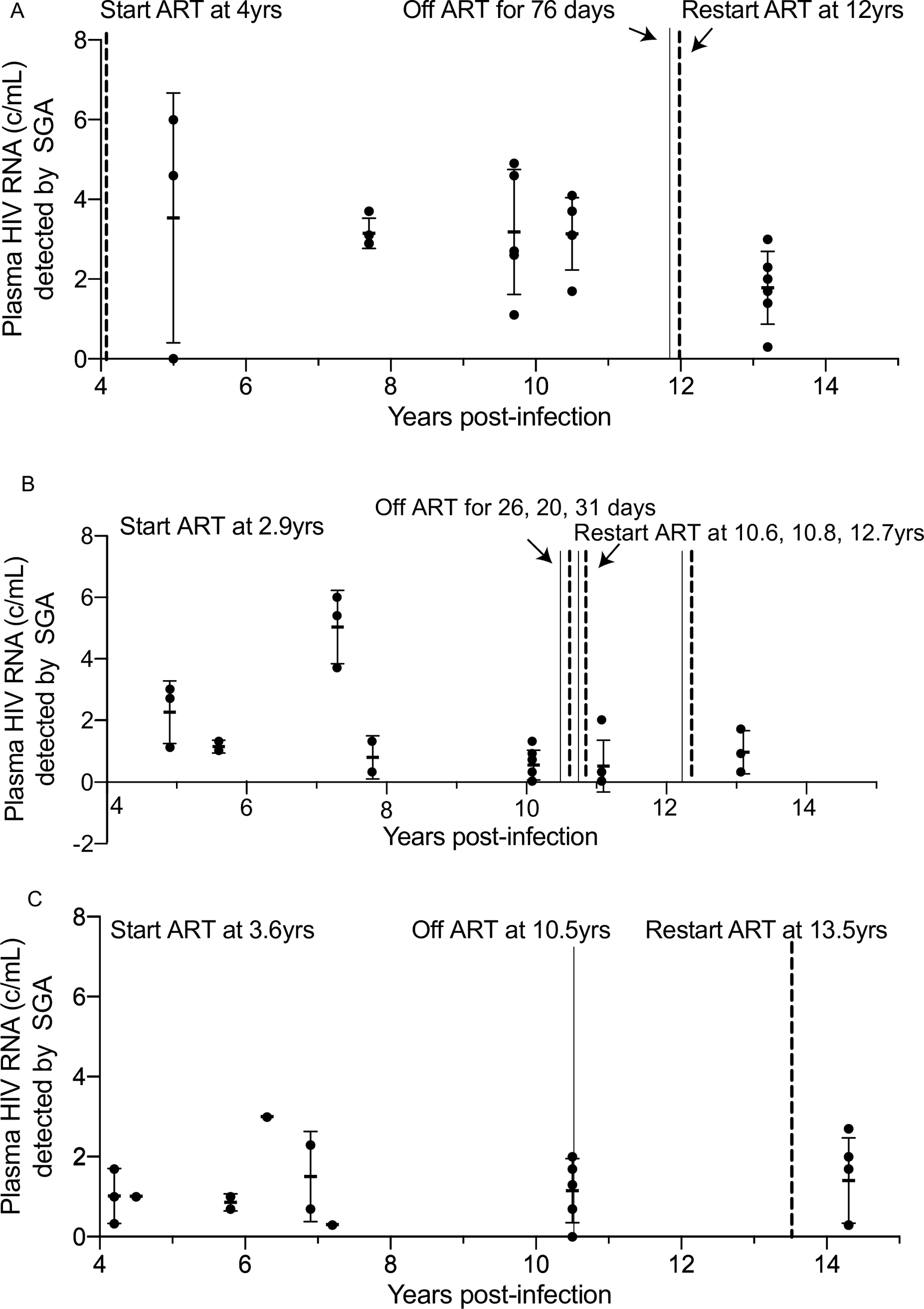

Supplement: S3 Fig — Each panel shows minimal concentration of virions (y-axis) estimated in specimens analyzed from Participants 1 (A), 2 (B), and 3 (C) at indicated timepoints (x-axis) following the estimated date of HIV infection. Each data point represents the total number of C2V5env sequences derived by SGA divided by the volume of each plasma aliquot, with means and standard deviations across aliquots from each timepoint shown. The time when ART was initiated or interrupted are shown with dotted and thin black vertical lines, respectively. (TIF) [file ppat.1008791.s003.tif]

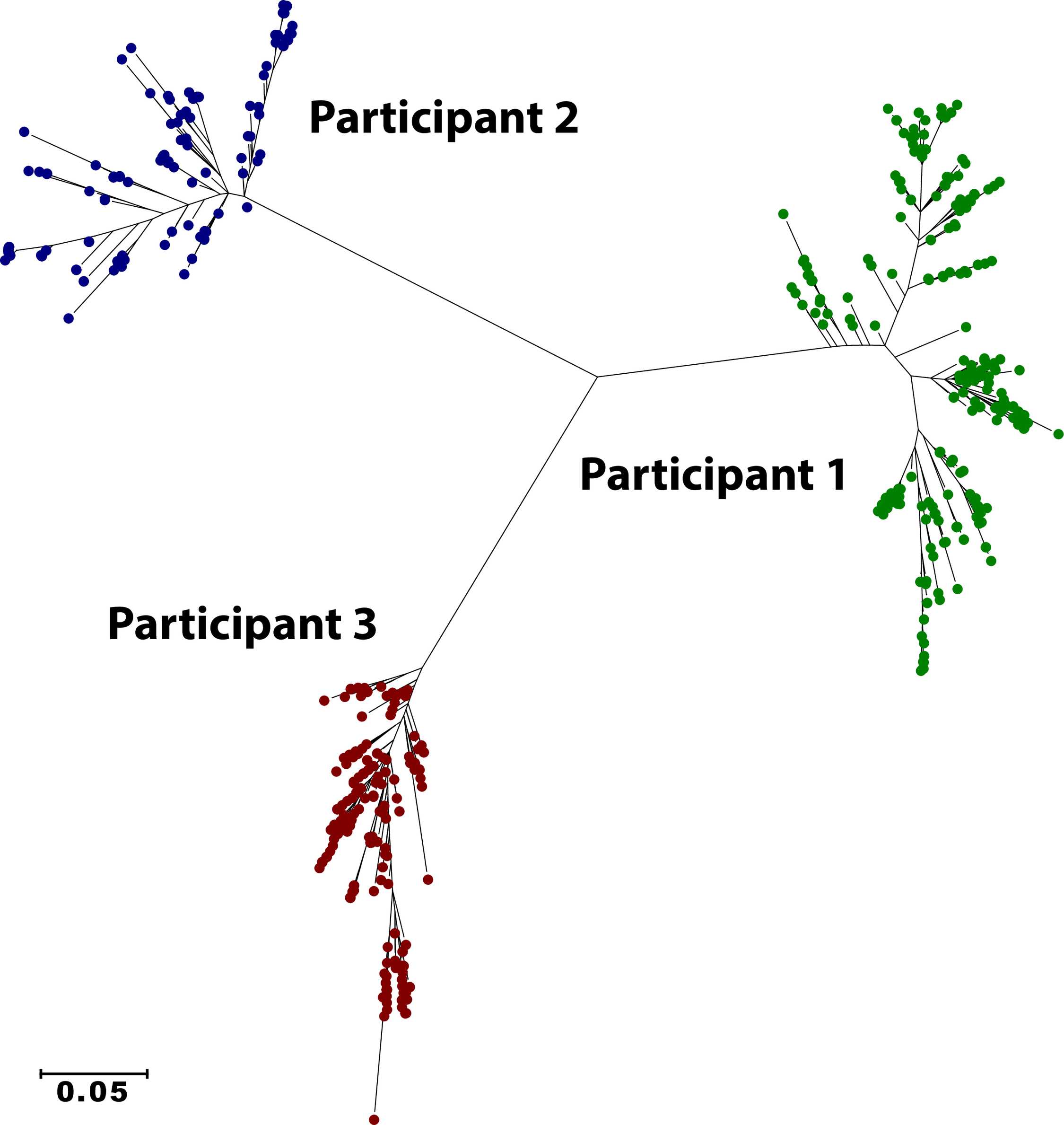

Supplement: S4 Fig — Participant’s 1–3 plasma and QVOA-derived single genome sequences are represented by green, blue and red, respectively, with each dot representing one sequence. A maximum likelihood phylogenetic tree of all 3 participants’ sequences shows segregation and clustering of sequences by participant indicating the lack of cross-contamination or sample mislabeling. (TIF) [file ppat.1008791.s004.tif]

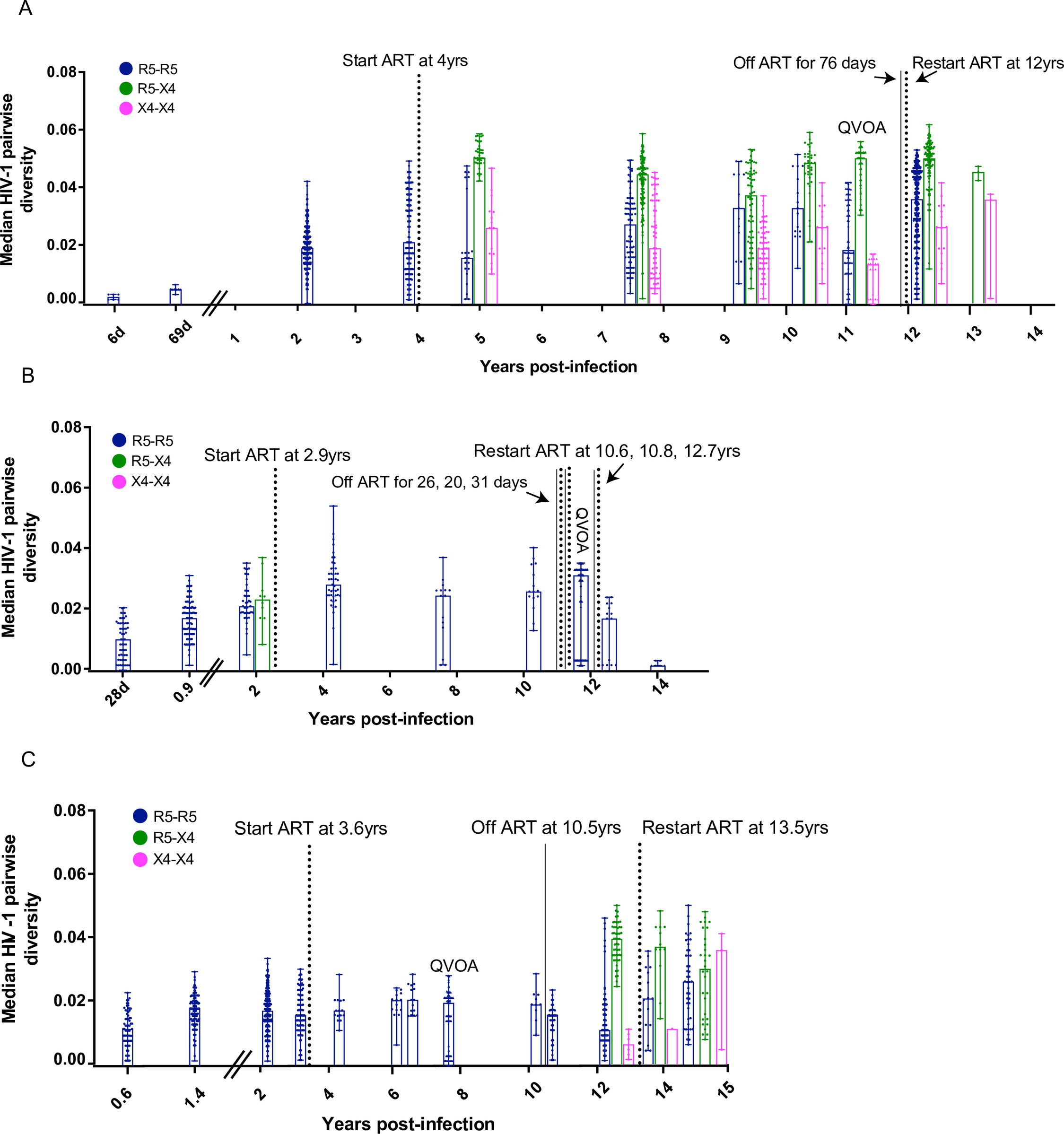

Supplement: S5 Fig — HIV-1 pairwise diversity within each specimen is shown with median and range of longitudinal unique plasma and QVOA-derived sequences for Participant 1 (A), Participant 2 (B), and Participant 3 (C). Colors represent comparisons between sequences from CCR5 vs. CCR5 (blue), CCR5 vs. dual/X4 (green), and dual/X4 vs. dual/X4 (magenta) tropisms. QVOA-derived sequences are indicated. The time when ART was initiated or interrupted are shown with dotted and thin black vertical lines, respectively. (TIF) [file ppat.1008791.s005.tif]

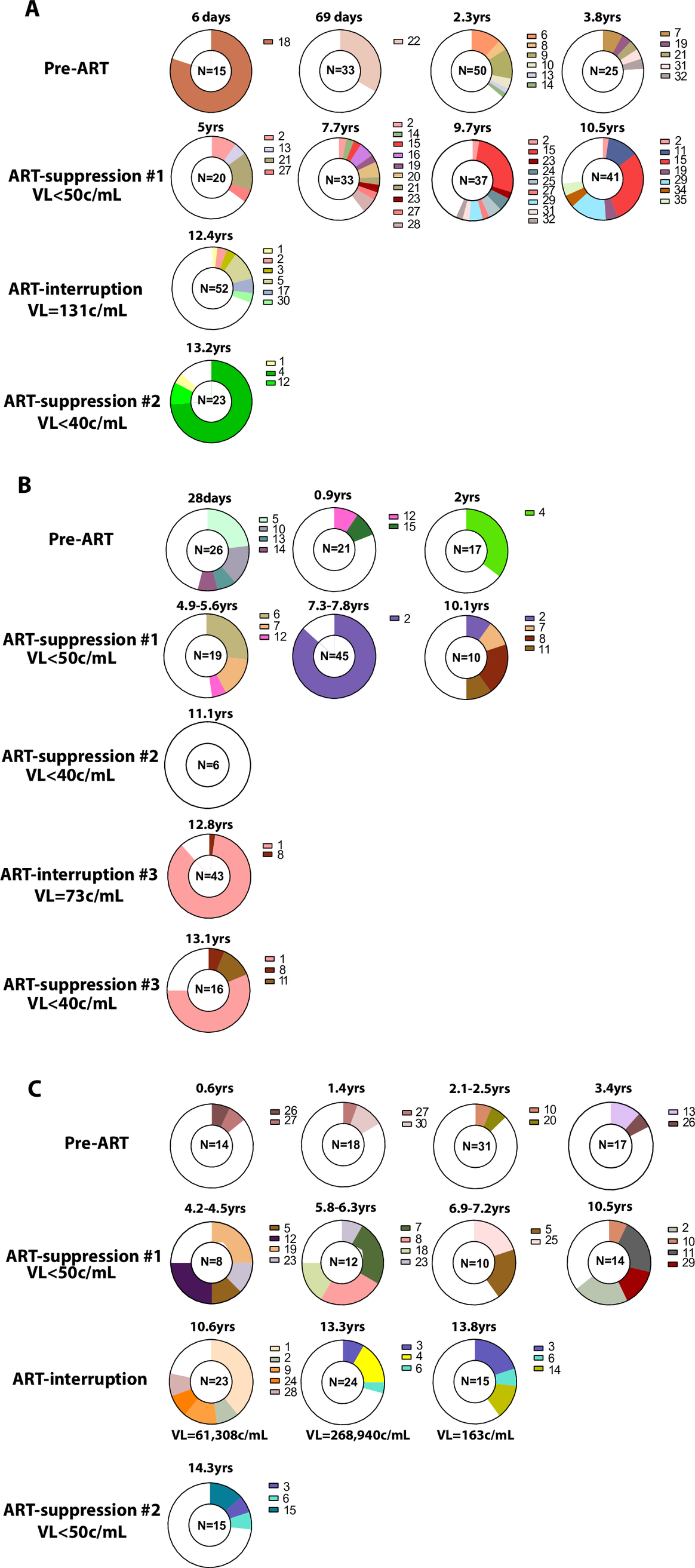

Supplement: S6 Fig — Each panel shows data for one participant: Participant 1 (A); Participant 2 (B); and Participant 3 (C). Each “donut” within the panels represents a timepoint with the segment colors representing identical variants at 1 or >1 timepoint. Shown is the proportion of HIV C2V5env sequences with each unique variant among all sequences amplified from that timepoint, with the total number of viral templates sequenced noted in the center of each donut. All variants detected only once are grouped in the white section of the donut. The time since the estimated date of HIV infection is indicated above the donut, and plasma HIV RNA load, if detectable, is noted either below the donut or to the left side of each panel. Variants detected in ≥2 specimens are shown with the same color. The donuts are organized horizontally within each panel by antiretroviral status, as described to the left of each panel. Numbers corresponding to each color in the key represent the specific variant with the same number and color maintained for persistent variants detected over time. (TIF) [file ppat.1008791.s006.tif]

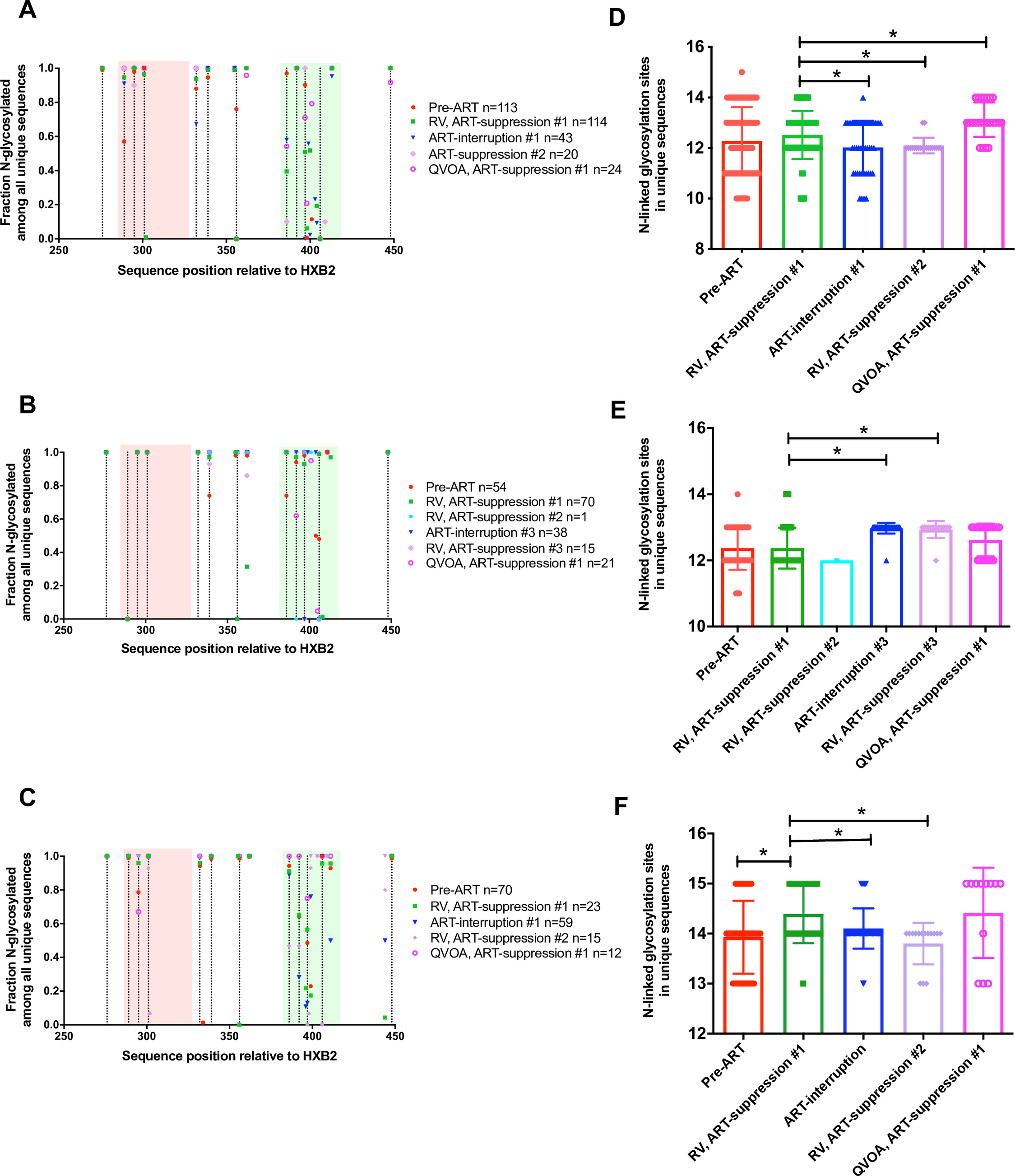

Supplement: S7 Fig — “N-glycosite” at Los Alamos National Laboratory (LANL) website (URL: https://www.hiv.lanl.gov/content/sequence/GLYCOSITE/glycosite.html; accessed on 9-12-19) was used to determine the fraction of N-linked glycosylation sites among all unique plasma and QVOA-derived C2V5env sequences from Participants 1 (A, D), 2 (B, E), and 3 (C, F). Panels A-C show the fraction of N-linked glycosylation sites (Y-axis) among unique sequences at positions mapped to the HIV reference genomes HXB2 (Genbank, K03455.1). Colors and symbols represent different plasma and QVOA timepoints. Dotted lines represent known HXB2 N-linked glycosylation sites (n = 12 sites) [67]. Red and green boxes represent the V3 and V4 regions in the HXB2 C2V5env sequence, respectively. Only unique C2V5env gene sequences that did not contain stop codons and that were not significantly G-A hypermutated are shown. Panels D-F show the mean +/- standard deviation of N-linked glycosylation sites among all unique plasma and QVOA sequences. The number of unique sequences included in the analysis is shown in the key. *p<0.05, 2-sample Wilcoxon rank sum test, adjusted for multiple comparisons using the Holm method, was performed to compare levels of glycosylation among RV during ART-suppression #1 relative to sequences from all other timeframes. (TIF) [file ppat.1008791.s007.tif]
